# Role of Phosphatidylinositol 4,5-Bisphosphate in Regulating EHD2 Plasma Membrane Localization

**DOI:** 10.1371/journal.pone.0074519

**Published:** 2013-09-10

**Authors:** Laura C. Simone, Steve Caplan, Naava Naslavsky

**Affiliations:** Department of Biochemistry and Molecular Biology and Eppley Cancer Center, University of Nebraska Medical Center, Omaha, Nebraska, United States of America; Purdue University, United States of America

## Abstract

The four mammalian C-terminal Eps15 homology domain-containing proteins (EHD1-EHD4) play pivotal roles in endocytic membrane trafficking. While EHD1, EHD3 and EHD4 associate with intracellular tubular/vesicular membranes, EHD2 localizes to the inner leaflet of the plasma membrane. Currently, little is known about the regulation of EHD2. Thus, we sought to define the factors responsible for EHD2’s association with the plasma membrane. The subcellular localization of endogenous EHD2 was examined in HeLa cells using confocal microscopy. Although EHD partner proteins typically mediate EHD membrane recruitment, EHD2 was targeted to the plasma membrane independent of two well-characterized binding proteins, syndapin2 and EHBP1. Additionally, the EH domain of EHD2, which facilitates canonical EHD protein interactions, was not required to direct overexpressed EHD2 to the cell surface. On the other hand, several lines of evidence indicate that the plasma membrane phospholipid phosphatidylinositol 4,5-bisphosphate (PIP2) plays a crucial role in regulating EHD2 subcellular localization. Pharmacologic perturbation of PIP2 metabolism altered PIP2 plasma membrane distribution (as assessed by confocal microscopy), and caused EHD2 to redistribute away from the plasma membrane. Furthermore, overexpressed EHD2 localized to PIP2-enriched vacuoles generated by active Arf6. Finally, we show that although cytochalasin D caused actin microfilaments to collapse, EHD2 was nevertheless maintained at the plasma membrane. Intriguingly, cytochalasin D induced relocalization of both PIP2 and EHD2 to actin aggregates, supporting a role of PIP2 in controlling EHD2 subcellular localization. Altogether, these studies emphasize the significance of membrane lipid composition for EHD2 subcellular distribution and offer new insights into the regulation of this important endocytic protein.

## Introduction

During endocytic transport, internalized molecules are routed along tubular/vesicular membranes where they are sorted for return to the cell surface, lysosomal degradation, or retrograde transport to the Golgi. Numerous regulatory proteins facilitate endocytic transport, among which are the four mammalian C-terminal Eps15 homology (EH) domain-containing proteins (EHD1-EHD4). EHD1, EHD2, EHD3 and EHD4 participate at distinct stages of trafficking. For instance, EHD1 regulates transport from the endocytic recycling compartment to the plasma membrane [1], [2], while EHD3 (the closest EHD1 paralog) mediates trafficking from early endosomes to the recycling compartment [3] or to the Golgi [4]. EHD4 localizes to early endosomes and regulates trafficking to the recycling compartment or to late endosomes/lysosomes [5], [6]. The EHDs are characterized by a C-terminal EH domain that binds to asparagine-proline-phenylalanine (NPF) motifs in partner proteins [7], [8]. The positively-charged electrostatic surface of these C-terminal EH domains leads to preferential binding with NPF motifs followed by acidic residues [9], [10]. The EHD N-terminus contains a G-domain that hydrolyzes ATP, flanked by two helical regions [11]. In the folded structure, the helical regions come together to form a coiled-coil domain that mediates EHD oligomerization [11]. Additionally, conserved lysine residues at positions 324, 327, 328 and 329, as well as a phenylalanine at position 322, facilitate EHD2 lipid binding [11].

Given that EHD1-EHD4 share 70–86% amino acid sequence identity, an important issue is the factors that determine the distinct subcellular localization and function of each EHD family member. EHD1, EHD3 and EHD4 are found on intracellular tubular/vesicular membranes. In contrast, EHD2 localizes to the cytoplasmic interface of the plasma membrane. Indeed, EHD2 is the most distinct of the four EHDs. While EHD1, EHD3 and EHD4 are capable of forming hetero-oligomers [6], [12],[13], EHD2 exclusively forms homo-oligomers [11]. As they lack a transmembrane domain, EHDs associate with membranes through specific protein and/or lipid interactions. For instance, localization of EHD1 to tubular recycling membranes depends on its interaction with the NPF motif-containing protein MICAL-L1 [14]. In addition, mutation of lysine 483 inhibits binding of EHD1 to phosphatidylinositol lipids, and induces localization of EHD1 to punctate rather than tubular membranes [15]. Thus, EHD1-lipid interactions are also important for regulating EHD1 subcellular localization.

Compared with EHD1, the regulation and function of EHD2 is less well characterized. Several studies now demonstrate that EHD2 associates with caveolae at the plasma membrane [16]–[19]. Caveolae are flask-shaped invaginations that are enriched in cholesterol [20], sphingolipids [21], [22], and phosphatidylinositol (4,5)-bisphosphate (PIP2) [23]. Oligomers of the integral membrane caveolin proteins form the framework of caveolae [24]. At the plasma membrane, association of multimeric complexes of soluble cavin proteins helps to shape and stabilize caveolar invaginations [25]–[27]. In addition, syndapin2 is present in a subset of caveolae, and is necessary for the formation of morphologically defined caveolae [17], [28], [29]. In the absence of either cavin1 [25] or syndapin2 [17], [28], [29], caveolin is distributed on flat regions of the plasma membrane. Caveolae are normally immobile structures [30], [31], but can undergo dynamic fusion/fission with the plasma membrane [32]. Recent studies ascribed a role for EHD2 in maintaining caveolae in a static state [18], [19]. Both Moren *et al*. and Stoeber *et al*. found that in the absence of EHD2, caveolae became more dynamic, evidenced by live cell imaging of caveolin1 [18], [19] and by electron microscopy of budded caveolae [18].

Although it is now well documented that EHD2 associates with caveolae, the mechanism by which EHD2 is recruited to these structures at the plasma membrane remains ambiguous. In the current study, we sought to define the factors that are necessary for EHD2 association with the plasma membrane. In doing so, we found an important role for the plasma membrane phospholipid PIP2 in regulating EHD2 subcellular localization. These findings lend important new information regarding the regulation of EHD2 and highlight its unique mode of recruitment to membranes compared with its close EHD paralogs.

## Results

### EHD2-specific Interacting Proteins are Dispensable for EHD2 Plasma Membrane Localization

**Figure 1:**
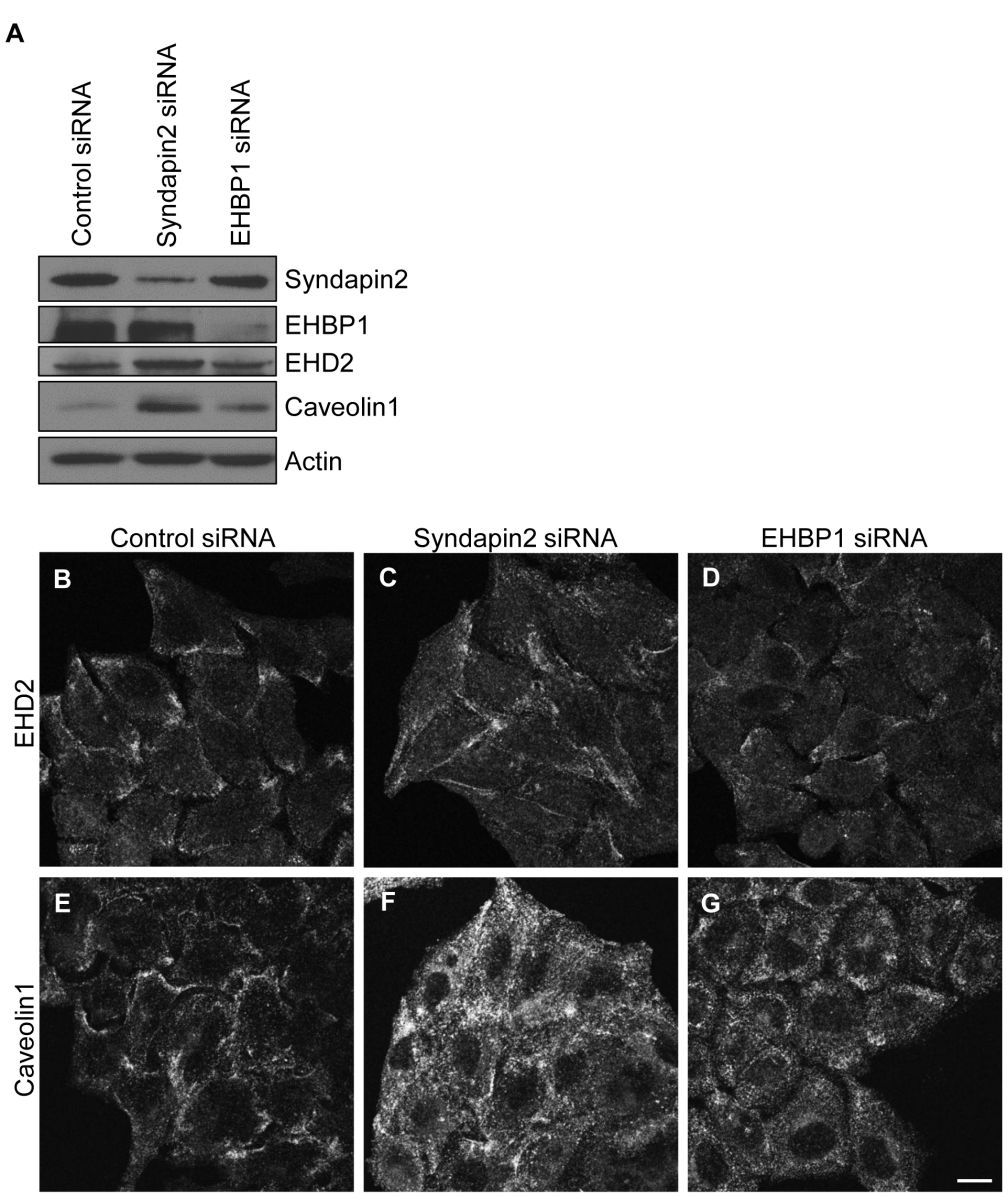
EHD2 localizes to the plasma membrane independent of its interaction partners, syndapin2 and EHBP1. HeLa cells were treated with non-targeting control-, syndapin2- or EHBP1-siRNA for 72 h. Cells were then lysed, and equal amounts of proteins were separated by SDS-PAGE and subjected to immunoblotting with antibodies against syndapin2, EHBP1, EHD2, caveolin1 or actin (A). For confocal microscopy, cells growing on coverslips were fixed and stained with antibodies against EHD2 (B–D) or caveolin1 (E–G). Bar, 10 µm. The immunoblots and confocal micrographs are representative of three independent experiments.

We first hypothesized that EHD2 subcellular localization is regulated by EHD2-binding proteins. Strong candidates for such proteins include syndapin2, a caveolae-localized protein that interacts directly with EHD2 [18], and the actin regulatory protein EHBP1 [33]. Both syndapin2 and EHBP1 interact with EHD2 via canonical EH domain-NPF motif binding. To determine the requirement of syndapin2 or EHBP1 for EHD2 subcellular localization, we knocked down either syndapin2 or EHBP1 in HeLa cells using siRNA. The efficiency of protein knockdown was verified by immunoblot (Fig. 1A). In addition to a significant decrease in syndapin2 expression, syndapin2-siRNA consistently induced enhanced EHD2 and caveolin1 protein levels (Fig. 1A, compare lanes 1 and 2). EHBP1 knockdown caused a slight increase in caveolin1 protein levels, but did not impact EHD2 protein expression (Fig. 1A, compare lanes 1 and 3).

We next assessed the subcellular localization of endogenous EHD2 in control-, syndapin2- and EHBP1-siRNA-treated HeLa cells. In control cells, endogenous EHD2 localized to small punctae that concentrated in patches along the cell edges (Fig. 1B). Endogenous caveolin1 exhibited a plasma membrane distribution similar to EHD2 (Fig. 1E), consistent with the known co-localization between EHD2 and caveolin1 [17]–[19]. Syndapin2- and EHBP1-siRNA altered EHD2 distribution, but did not prevent EHD2 plasma membrane localization. Syndapin2-knockdown caused an increase in the amount of punctate EHD2 (Fig. 1C) and caveolin1 (Fig. 1F) at the plasma membrane (especially for caveolin1), both within patches and across the plasma membrane. The increased plasma membrane localization of EHD2 and caveolin1 may be related in part to the elevated total levels of these proteins (Fig. 1A). Depletion of EHBP1 caused a partial dispersal of EHD2 (Fig. 1D) and caveolin1 (Fig. 1G) to more homogeneously spread punctae. Although EHD2 was partially in plasma membrane patches, EHD2 and caveolin1 punctae were dispersed across the plasma membrane in the majority of EHBP1-siRNA-treated cells. Overall, these data demonstrate that neither syndapin2 nor EHBP1 are necessary for localization of EHD2 to the plasma membrane.

**Figure 2:**
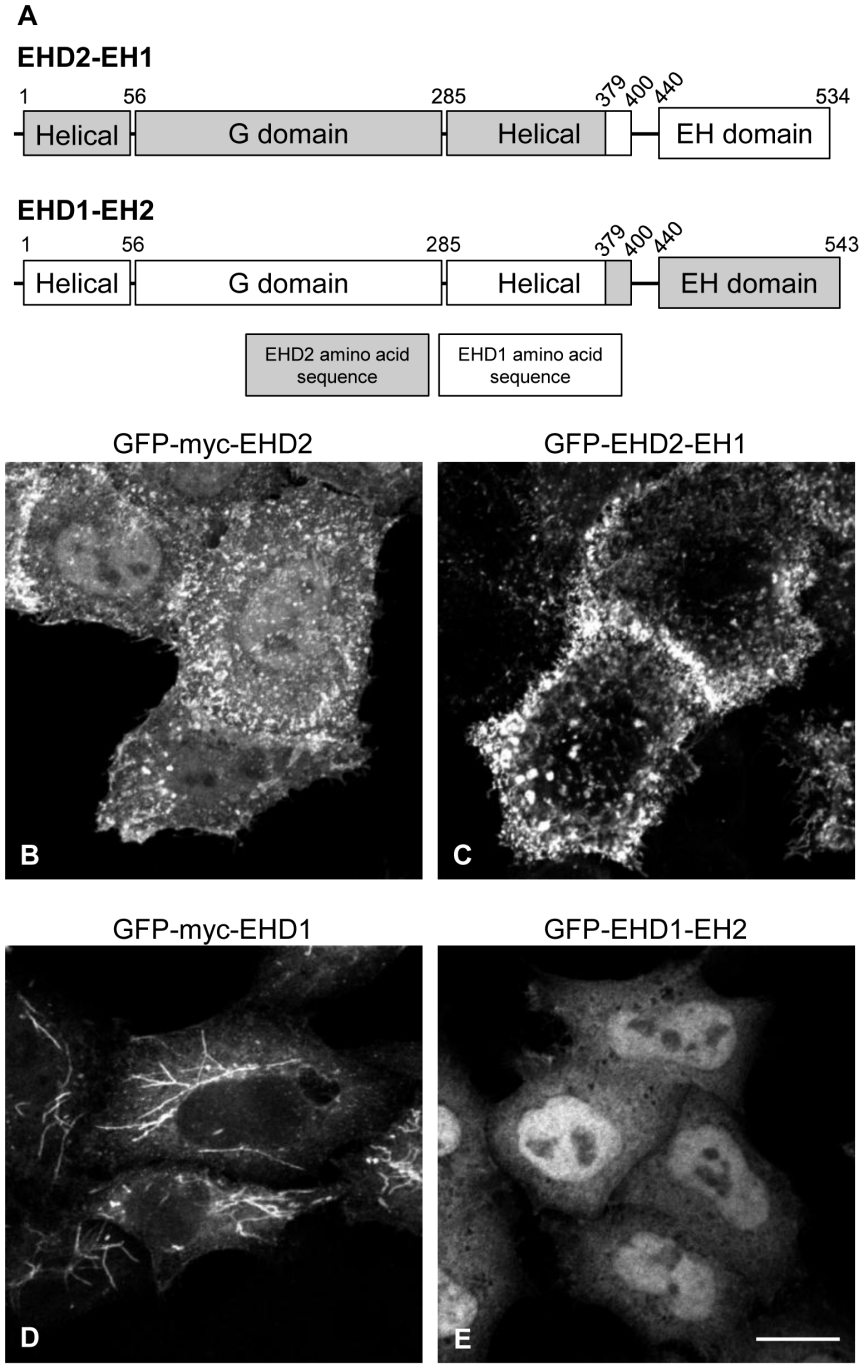
Unlike EHD1, membrane targeting of EHD2 is independent of its EH domain. (A) Schematic representation of EHD chimeras. The EHD2-EH1 chimera contains EHD2 amino acids 1–378 (shown in grey) and EHD1 amino acids 379–534 (encoding the EH domain, shown in white). Conversely, the EHD1-EH2 chimera contains EHD1 amino acids 1–378 (shown in white) and EHD2 amino acids 379–543 (encoding the EH domain, shown in grey). HeLa cells growing on coverslips were transfected with GFP-Myc-EHD2 (B), GFP-EHD2-EH1 (C), GFP-Myc-EHD1 (D), or GFP-EHD1-EH2 (E). After 24 h, the cells were fixed and analyzed directly for GFP-fusion protein expression by confocal microscopy. Bar, 10 µm. The confocal micrographs are representative of two independent experiments.

To address the extent to which other undefined protein interactions might influence EHD2 plasma membrane localization, we generated chimeras of EHD2 and EHD1 in which the EH domains were swapped. We reasoned that if protein interactions mediated through the EH2 domain are responsible for EHD2 plasma membrane localization, then replacement with the EH1 domain should alter the subcellular distribution of the chimera. To this end, we made an EHD2-EH1 chimera in which the EHD2 EH domain was exchanged for the EHD1 EH domain (Fig. 2A). Conversely, the EHD1-EH2 chimera comprised amino acids 1–378 of EHD1 fused to the EHD2 C-terminus (Fig. 2A). Myc-tagged wild type EHD2 and EHD1 as well as the EHD2-EH1 and EHD1-EH2 chimeras were cloned into an EGFP vector and overexpressed in HeLa cells. As shown in Fig. 2B, GFP-Myc-EHD2 was found in large punctae at the plasma membrane. The GFP-EHD2-EH1 chimera also displayed a punctate, plasma membrane pattern (Fig. 2C), indicating that targeting of EHD2 to the plasma membrane is not governed by interactions involving the EH2 domain. In contrast, while GFP-Myc-EHD1 displayed characteristic tubular localization (Fig. 2D), the GFP-EHD1-EH2 chimera neither associated with recycling tubules nor with the plasma membrane; instead, its localization was primarily cytosolic and nuclear (Fig. 2E). The presence of recycling tubules in GFP-EHD1-EH2-overexpressing HeLa cells was verified by immunostaining with MICAL-L1, a marker of recycling tubules (data not shown). Thus, unlike EHD2, subcellular targeting of EHD1 to membranes depends on amino acid sequence information contained in the EH1 domain.

### PIP2 Regulates EHD2 Plasma Membrane Localization

**Figure 3:**
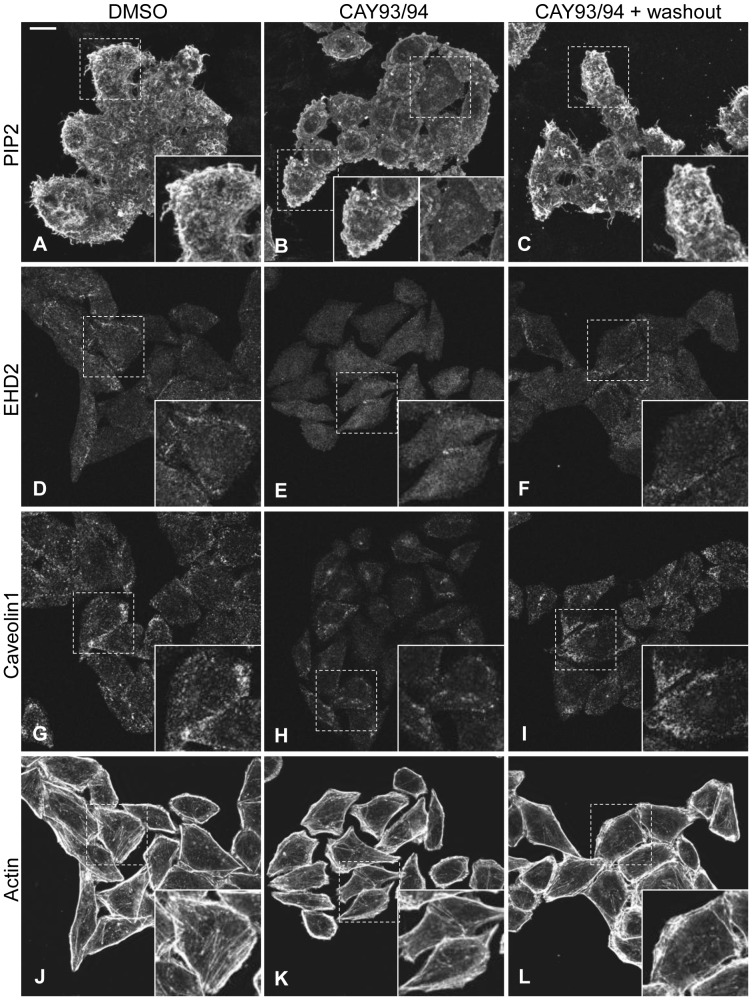
Inhibition of PLD alters PIP2 distribution and results in the loss of EHD2 and caveolin1 from the plasma membrane. HeLa cells growing on coverslips were treated with DMSO or with 25 µM each of the PLD inhibitors CAY10593 and CAY10594 (CAY93/94) for 45 min. The cells were then fixed or washed 3X with DMEM and chased in complete growth medium for 2 h (CAY93/94+ washout). After fixation, the cells were stained with purified GST-PH-PLCδ1 to visualize PIP2 (A–C), or with antibodies against EHD2 (D–F) or caveolin1 (G–I), or with phalloidin-488 to visualize actin (J–L). After incubation with the appropriate primary and secondary antibodies, the cells were analyzed by confocal microscopy. The images showing PIP2 staining (A–C) are z-series stacks, while cells stained for EHD2, caveolin1 or actin (D–L) were imaged as single optical sections. The images shown in D–F and J–L are the same set of cells that were co-stained for EHD2 and actin. Bar, 10 µm. The confocal micrographs are representative of three independent experiments.

Since EHD2 plasma membrane targeting did not depend on protein interactions mediated through the EH2 domain, we asked whether specific phospholipids might recruit EHD2 to the plasma membrane. PIP2 is enriched at the plasma membrane, and EHD2 binds to PIP2 *in vitro* [11], [34]. However, the role of PIP2 in regulating EHD2 *in vivo* has not been explored. To address this issue, we used specific inhibitors of phospholipase D (PLD). PLD-derived phosphatidic acid (PA) activates phosphatidylinositol 4-phosphate (PI4P) 5-kinase, which converts PI4P to PIP2 [35]. Therefore, inhibiting PLD indirectly impacts PIP2 generation. CAY10593 and CAY10594 inhibit the two mammalian PLD isoforms PLD1 and PLD2, respectively [36]. To visualize PIP2, we employed an innovative approach developed by Hammond *et al*. [37] wherein cells were stained with the glutathione S-transferase (GST)-tagged pleckstrin homology (PH) domain of phospholipase C δ1 (PLCδ1), which binds with high affinity to PIP2. Using this method, we found that in control DMSO-treated cells, PIP2 localized to the plasma membrane and displayed a concentration in filopodia-like structures (Fig. 3A). Treatment with CAY10593 and CAY10594 (CAY93/94) induced two PIP2 phenotypes. In several of the CAY93/94-treated cells, PIP2 no longer localized to distinct plasma membrane-associated structures (Fig. 3B, right insert). In this subpopulation of treated cells, PIP2 levels were reduced on the plasma membrane. Alternatively, some of the CAY93/94-treated cells displayed plasma membrane blebs containing PIP2 (Fig. 3B, left insert). Filopodia could be seen (albeit faintly) by staining actin in DMSO-treated cells (data not shown). Treatment with CAY93/94 greatly reduced filopodia; instead, the membrane blebs were apparent (data not shown). Cellular blebbing occurs in several physiological contexts that require cell detachment, including migration, cell division and apoptosis [38]. Cells with PIP2 blebs were rounded, suggesting that the PLD inhibitors were affecting cell adhesion. Indeed, treatment with CAY93/94 prevented HeLa cells from attaching to fibronectin-coated coverslips (data now shown), consistent with previous findings that PIP2 generated downstream of PLD is important for integrin-mediated cell adhesion [39]. In total, these data demonstrate that treatment with CAY93/94 impacts PIP2 distribution/levels.

Importantly, CAY93/94 caused a marked relocalization of both EHD2 and caveolin1 away from the plasma membrane (compare Fig. 3D to 3E, and Fig. 3G to 3H; see insets). Removal of CAY93/94 restored PIP2 to the plasma membrane and filopodia (Fig. 3C), and facilitated recovery of EHD2 (Fig. 3F) and caveolin1 (Fig. 3I) to the plasma membrane. These data strongly support a role for PIP2 in EHD2 (as well as caveolin1) maintenance at the plasma membrane. CAY93/94 did not dramatically alter the actin cytoskeleton (Fig. 3J–L), although these data do not rule out subtle changes in cortical/filamentous actin that may be associated with alterations in cell adhesion. These data suggest that EHD2 and caveolin1 relocalization was primarily due to changes in plasma membrane PIP2 levels, as opposed to cytoskeletal changes.

**Figure 4:**
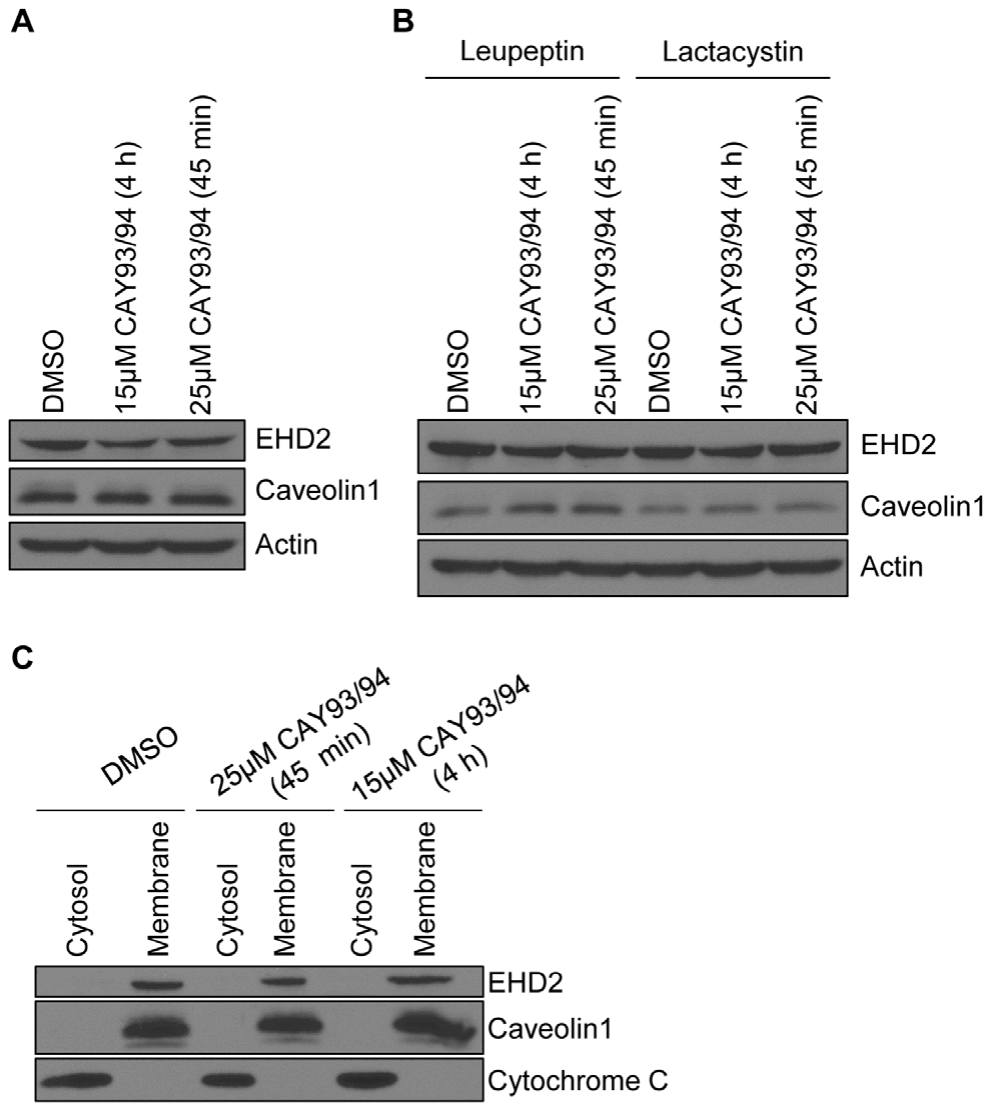
Acute PLD inhibition reduces EHD2 protein levels, but does not dissociate EHD2 from membranes. (A) HeLa cells were treated with DMSO (control), or 15 µM each CAY10593 and CAY10594 for 4 h, or 25 µM each CAY10593 and CAY10594 for 45 min. The cells were then lysed, and equal amounts of proteins were separated by SDS-PAGE and subjected to immunoblotting with antibodies against EHD2, caveolin1 or actin. The immunoblots are representative of seven independent experiments. (B) HeLa cells were pre-treated with 100 µM leupeptin or with 10 µM lactacystin for 3 h. The cells were then treated with fresh 100 µM leupeptin or 10 µM lactacystin along with either DMSO, 15 µM each CAY10593 and CAY10594 (for 4 h), or 25 µM each CAY10593 and CAY10594 (for 45 min). Following treatment, the cells were lysed, and equal amounts of proteins were separated by SDS-PAGE and subjected to immunoblotting with antibodies against EHD2, caveolin1 or actin. The immunoblots are representative of three experiments conducted with leupeptin, and two experiments done with lactacystin. (C) HeLa cells were treated as in (A). The cells were then homogenized and post-nuclear supernatants were separated into cytosol and membrane fractions by ultracentrifugation. The samples were resolved by SDS-PAGE and immunoblotted with antibodies against EHD2, caveolin1 or cytochrome C (cytosolic fraction control). The immunoblots are representative of two independent experiments.

The finding that EHD2 and caveolin1 returned to the plasma membrane within two hours of CAY93/94 washout suggested that EHD2/caveolin1 were not undergoing substantial protein degradation. Indeed, immunoblot analysis showed that caveolin1 levels were unchanged during the 45 minute CAY93/94 treatment (Fig. 4A, compare lanes 1 and 3). Even when cells were treated for a prolonged period with a lower concentration of CAY93/94 (4 hours with 15 µM CAY93/94), caveolin1 levels remained constant (Fig. 4A, compare lanes 1 and 2). EHD2 protein levels, on the other hand, were slightly decreased by either CAY93/94 regimen (Fig. 4A, compare lane 1 with lanes 2 and 3). Treatment with the lysosomal inhibitor leupeptin or the proteasomal inhibitor lactacystin led to a partial rescue of EHD2 levels in the presence of CAY93/94 (Fig. 4B), suggesting that a portion of EHD2 is targeted for degradation upon dislocation from the plasma membrane. Notably, proteasome activity can contribute to lysosomal degradation [40], which may explain the effect of both leupeptin and lactacystin on EHD2 levels. Cellular fractionation revealed that both caveolin1 and EHD2 were maintained on membranes upon CAY93/94 treatment (Fig. 4C). Thus, even when displaced from the plasma membrane by CAY93/94, EHD2 and caveolin1 did not become cytosolic. Overall, these studies indicate that PLD-derived PIP2 is necessary for maintaining EHD2 at the plasma membrane.

**Figure 5:**
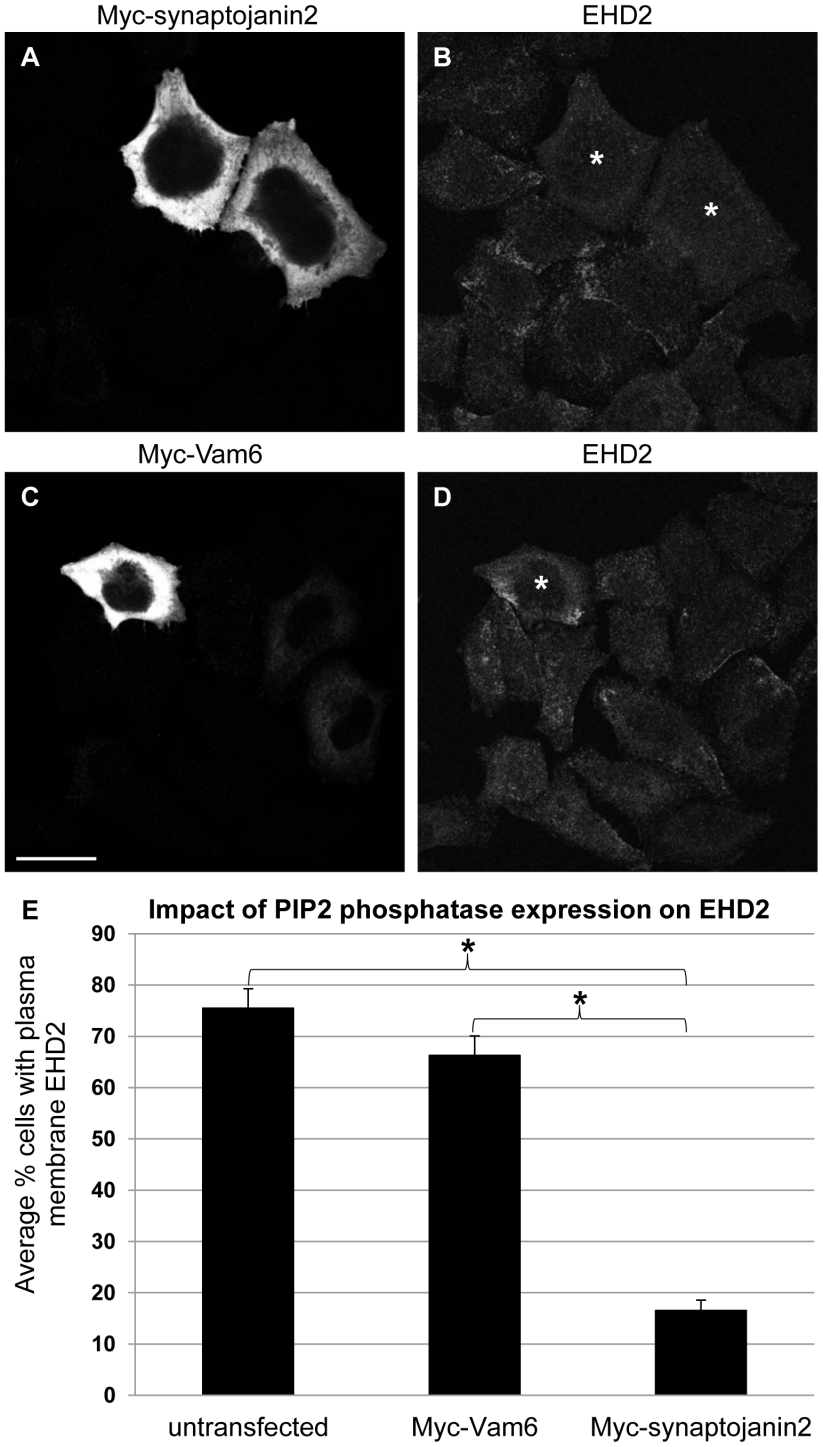
Expression of the PIP2 phosphatase Myc-synaptojanin2 reduces EHD2 at the plasma membrane. HeLa cells growing on coverslips were transfected with Myc-synaptojanin2 (A–B) or with Myc-Vam6 (C–D). After 24 h, the cells were fixed and stained with antibodies against Myc and EHD2. Following incubation with fluorochrome-conjugated secondary antibodies, the cells were analyzed by confocal microscopy. Bar, 10 µm. Transfected cells are marked with an asterisk. The confocal micrographs are representative of three independent experiments. (E) The number of untransfected, Myc-Vam6-transfected (control), or Myc-synaptojanin2-transfected cells that displayed EHD2 at the plasma membrane was counted. A total of at least 50 untransfected or transfected cells from 3 independent experiments were counted. The average percentage of cells displaying EHD2 at the plasma membrane was compared between the groups by performing a Tukey HSD test (*p<0.01).

As an additional means of manipulating PIP2 levels *in vivo*, HeLa cells were transfected with the 5-phosphatase Myc-synaptojanin2, which hydrolyzes PIP2 [41], [42]. As shown in Fig. S2, expression of Myc-synaptojanin2 caused a pronounced decrease in PIP2 levels. Next, the impact of Myc-synaptojanin2 expression on EHD2 subcellular localization was assessed by confocal microscopy. To control for effects from the transfection process, HeLa cells were transfected with Myc-Vam6, which acts at late endosomes/lysosomes [43], and is not expected to impact lipids at the plasma membrane. Quantification revealed that 75.5% (±3.8%) of untransfected cells displayed punctate EHD2 at the plasma membrane (Fig. 5E). Transfection with Myc-Vam6 did not significantly impact localization of EHD2 to the plasma membrane (Fig. 5C–E). In contrast, transfection with Myc-synaptojanin2 induced a striking decrease in EHD2 plasma membrane localization (Fig. 5A–B). Indeed, only 16.5% (±2.0%) of cells expressing Myc-synaptojanin2 retained visible EHD2 at the cell surface (Fig. 5E). This finding provides strong support for the hypothesis that PIP2 is required to maintain EHD2 at the plasma membrane.

**Figure 6:**
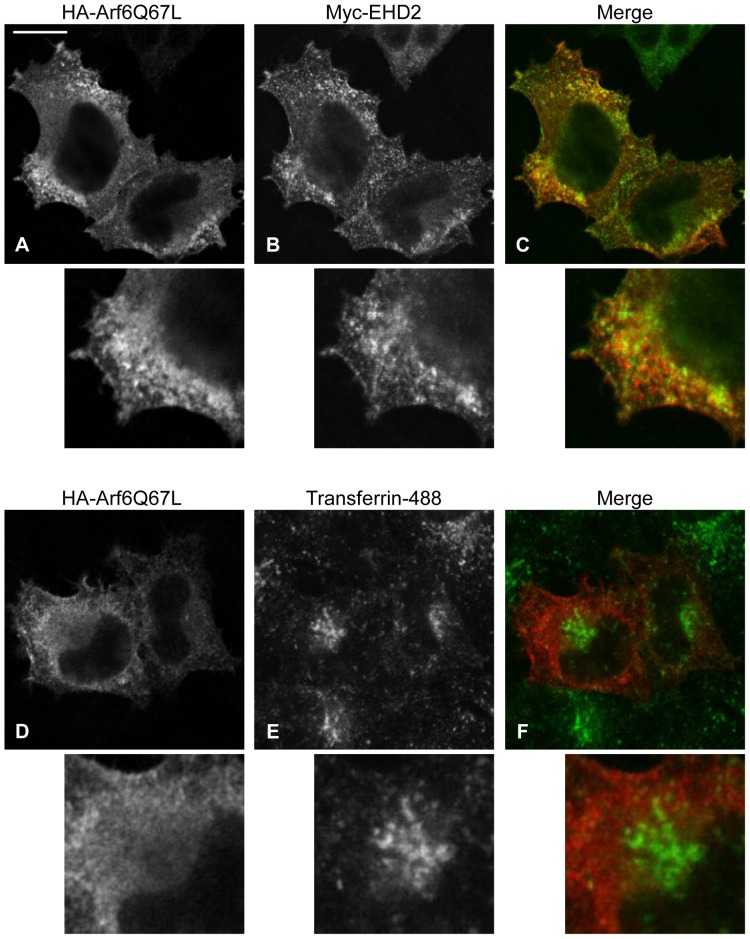
EHD2 localizes to PIP2-enriched Arf6Q67L-positive vacuoles. (A–C) HeLa cells growing on coverslips were transfected with HA-Arf6Q67L and Myc-EHD2 for 11 h. The cells were then fixed and stained with antibodies against HA and Myc. After addition of fluorochrome-conjugated secondary antibodies, the cells were analyzed by confocal microscopy. (D–E) HeLa cells growing on coverslips were transfected with HA-Arf6Q67L for 11 h. The cells were incubated in serum-free DMEM containing 0.5% bovine serum albumin during the last 3.5 h of transfection, and then pulsed with 1 µg/ml transferrin-488 for 20 min. The cells were then fixed and stained with antibody against HA. After incubation with fluorochrome-conjugated secondary antibody, the cells were analyzed by confocal microscopy. Insets are shown below each image. Bar, 10 µm. The confocal micrographs are representative of three independent experiments.

To further address the importance of PIP2 for EHD2 subcellular localization, we used a constitutively-active Arf6 mutant (Arf6Q67L). Overexpression of Arf6Q67L activates PI4P 5-kinase and induces plasma membrane-derived vacuoles enriched in PIP2 [44]. We reasoned that if PIP2 regulates EHD2 subcellular localization, then EHD2 should be recruited to PIP2-enriched vacuoles in HA-Arf6Q67L-expressing cells. Indeed, when HA-Arf6Q67L and Myc-EHD2 were co-expressed, Myc-EHD2 was readily detected in the HA-Arf6Q67L-induced vacuoles (Fig. 6A–C, see magnifications). In contrast, transferrin receptor, which does not bind PIP2, remained distinct from HA-Arf6Q67L-containing structures (Fig. 6D–F, see magnifications). Thus, EHD2 appears to maintain its association with PIP2 as it is detached from the plasma membrane into Arf6-containing vacuoles.

### EHD2 Localization to the Plasma Membrane is Independent of Actin Microfilaments

**Figure 7:**
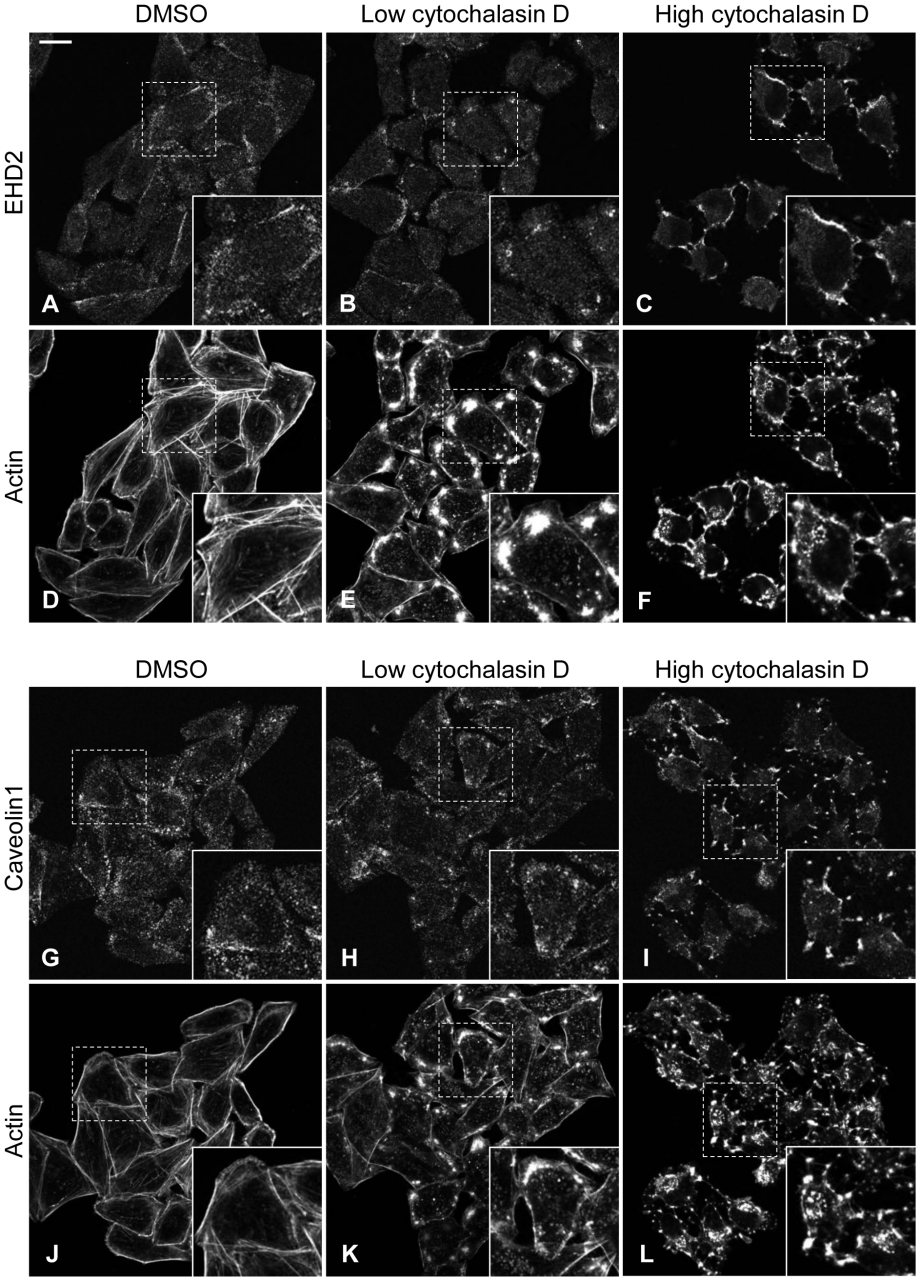
EHD2 and caveolin1 localization to the plasma membrane is independent of intact actin microfilaments. HeLa cells growing on coverslips were treated with DMSO, or with a low (0.3 µM) or high (5 µM) concentration of cytochalasin D for 45 min. The cells were then fixed and stained with antibodies against EHD2 (A–C) or caveolin1 (G–I) along with phaolloidin-488 to visualize actin (D–F, J–L). After incubation with fluorochrome-conjugated secondary antibody, the cells were analyzed by confocal microscopy. Insets depict the regions in the dashed boxes. Bar, 10 µm. The confocal micrographs are representative of three independent experiments with 0.3 µM cytochalasin D, and two independent experiments with 5 µM cytochalasin D.

Local synthesis and metabolism of PIP2 at the plasma membrane is crucial for actin regulation. The actin cytoskeleton is intimately tied to PIP2 through actin regulatory proteins that also bind to PIP2 (e.g. Wiskott Aldrich syndrome protein, cofilin, vinculin, etc.) [45]. Stoeber *et al*. suggested that EHD2 provides a link between caveolae and the actin cytoskeleton [19]. Hence, we sought to determine the involvement of actin in maintaining EHD2 at the plasma membrane. Treatment with a low concentration of cytochalasin D disrupted actin microfilaments (compare Fig. 7D to 6E, and 6J to 6K). Yet, EHD2 and caveolin1 were generally retained at the plasma membrane (compare Fig. 7A to 7B, and Fig. 7G to 7H). At this low dose of cytochalasin D, EHD2 and caveolin1 began to redistribute to sites of actin aggregation (compare insets in Fig. 7B with 7E, and Fig. 7H with 7K). Even at a high concentration of cytochalasin D, which completely eliminated actin filaments and cortical actin, EHD2 persisted at the plasma membrane, but was now entirely concentrated in actin-containing aggregates (Fig. 7C and 7F, see insets). Similarly, caveolin1 localized to sites of actin aggregation upon treatment with the high dose of cytochalasin D (Fig. 7I and 7L, see insets). Immunoblot analysis showed that neither EHD2 nor caveolin1 levels were altered by treatment with the low or high dose of cytochalasin D (data not shown).

**Figure 8:**
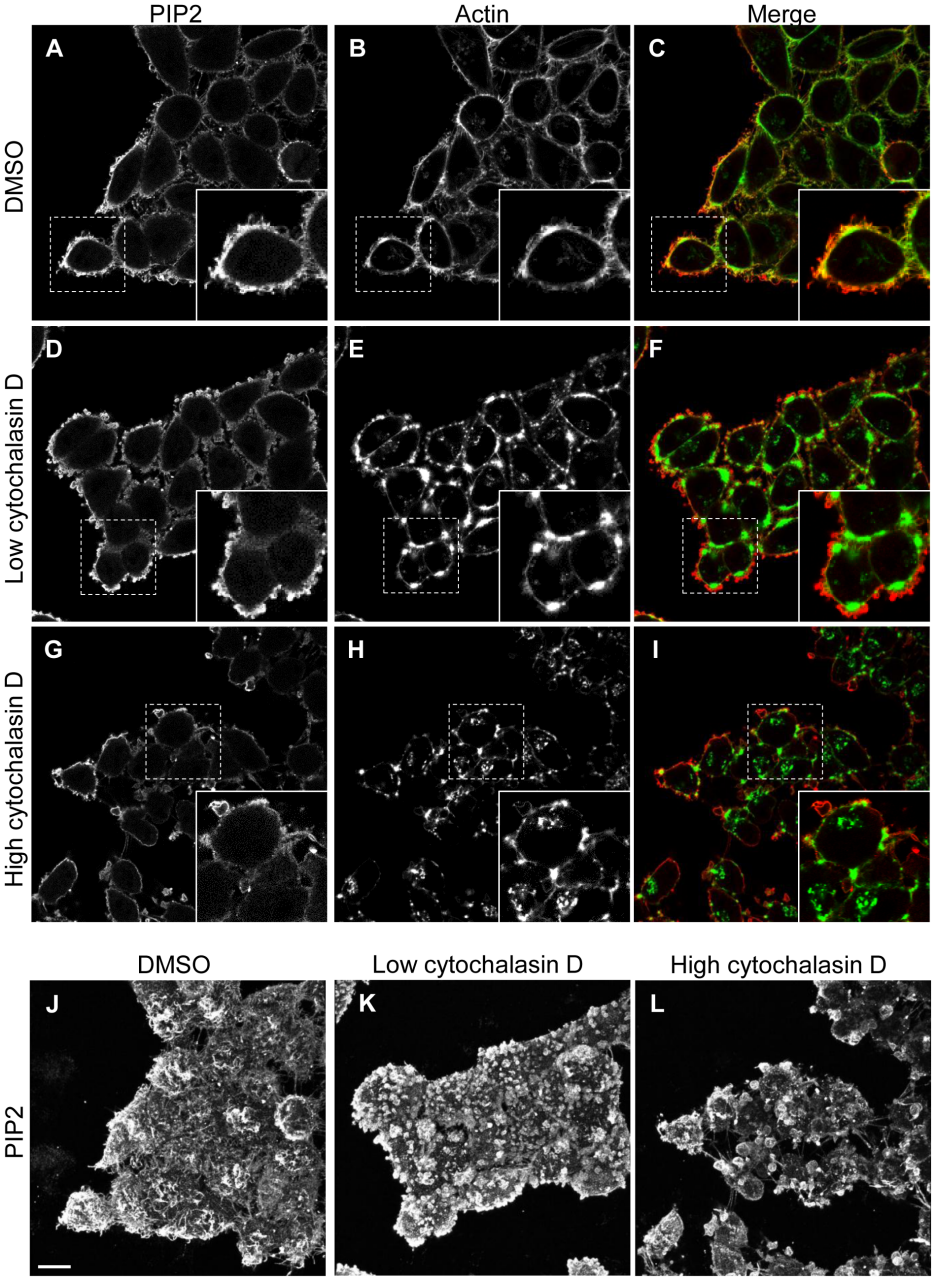
Cytochalasin D alters PIP2 distribution. HeLa cells growing on coverslips were treated with DMSO, or with a low (0.3 µM) or high (5 µM) concentration of cytochalasin D for 45 min. The cells were then fixed and stained with purified GST-PH-PLCδ1 to visualize PIP2 (A, D, G, J-K) along with phalloidin-488 to visualize actin (B, E, H). For the PIP2 immunofluorescence, the cells were next stained with antibody against GST and fluorochrome-conjugated secondary antibody. Cells were then analyzed by confocal microscopy. The images shown in A–I are single optical sections taken near the ventral cell surface, whereas the images shown in J–K are z-series stacks. Bar, 10 µm. The confocal micrographs are representative of three independent experiments.

Not surprisingly, given the close connection between PIP2 and actin, cytochalasin D affected PIP2 distribution. In control DMSO-treated cells, PIP2 overlaid with cortical actin at the plasma membrane, and coalesced with filamentous actin in filopodia-like structures (Fig. 8A–C). Treatment with the low dose of cytochalasin D induced extensive PIP2-containing membrane blebs that surrounded cytochalasin-induced actin aggregates (Fig. 8D–F). In the high dose cytochalasin D-treated cells, PIP2 was found in a thin, flat layer at the membrane and on large blebs (Fig. 8G–I). Overall, cytochalasin D altered PIP2 distribution at the plasma membrane, but did not noticeably diminish PIP2 levels (Fig. 8J–L). In sum, these data indicate that preservation of an intact actin cytoskeleton is dispensable for the plasma membrane localization of EHD2 and caveolin1. However, the organization of the actin cytoskeleton clearly impacts EHD2 and caveolin1 distribution within the plasma membrane.

## Discussion

EHD2’s localization to caveolae and the inner leaflet of the plasma membrane sets it apart from the other EHD proteins, and poses the question as to what drives its unique subcellular distribution. Our data establish PIP2 as a crucial factor in maintaining EHD2 at the plasma membrane. We show that EHD2 is recruited to PIP2-enriched membranes formed by Arf6Q67L in cells (Fig. 6), consistent with EHD2’s *in vitro* PIP2 binding ability [11], [34]. Furthermore, manipulating PIP2 levels *in vivo* by inhibiting PLD or by expression of synaptojanin2 caused a dramatic relocalization of EHD2 away from the plasma membrane (Fig. 3 and Fig. 5). Powner, *et al*. found that PLD-derived PIP2 promotes integrin-dependent cell adhesion [39], and work by del Pozo, *et al*. showed that caveolae are rapidly internalized upon loss of integrin-mediated attachment [46]. Thus, a potential mechanism for EHD2 plasma membrane displacement upon CAY93/94 treatment is through loss of integrin-mediated adhesion and subsequent caveolar internalization. As such, our studies also indicate that PIP2 is important in maintaining caveolae at the cell surface.

It should also be noted that our data do not exclude the possibility that PA might play a minor role in linking EHD2 to the plasma membrane. Indeed, our recent work shows that two EHD1-interacting proteins, MICAL-L1 and syndapin2, bind to PA on tubular recycling endosomes [47]. However, in contrast to EHD1, EHD2 membrane recruitment occurs independently of syndapin2 (and its binding to PA) (Fig. 1C). In simple fractionation experiments, EHD2 showed binding to PIP2, and to other phosphorylated inositol groups [34], but to our knowledge, direct binding of EHD2 to PA has not been tested. However, given that EHD2 binds to PIP2 *in vitro* [11], and PIP2 is enriched at the plasma membrane, it is likely that the effects of PLD inhibition and synaptojanin2 expression on EHD2 localization primarily result from PIP2 binding. In further support of this conclusion, our confocal micrographs show that the PLD inhibitors CAY93/94 and synaptojanin2 expression dramatically impact PIP2 at the plasma membrane (Fig. 3A–C and Fig. S2).

The EHD1 partner proteins MICAL-L1 and syndapin2 cooperate in recruiting EHD1 to tubular recycling endosomes [14], [47]. In contrast, we found that neither syndapin2 nor EHBP1 are necessary to recruit and/or maintain EHD2 at the plasma membrane (Fig. 1). Furthermore, replacing the EH2 domain with the EH1 domain (in the GFP-EHD2-EH1 chimera) did not impact EHD2 subcellular localization (Fig. 2). This finding indicates that NPF motif-containing EHD2 partners are not critical for EHD2 plasma membrane recruitment. Along these lines, George *et al*. and Moren *et al*. found that EHD2ΔEH (lacking the EH domain) maintained a distribution similar to wild type EHD2 [5], [18], leading Moren *et al*. to conclude that the EHD2-syndapin2 interaction is not necessary for targeting EHD2 to caveolae. Importantly, our syndapin2-siRNA studies allow us to draw additional, informative conclusions regarding EHD2 subcellular regulation. For instance, our finding that EHD2 remains at the plasma membrane upon syndapin2 knockdown (Fig. 1C), which impedes caveolar invagination [17], [28], argues that morphologically defined caveolae are not necessary for EHD2 recruitment. Additionally, syndapin2 knockdown enhanced the total protein levels of EHD2 and caveolin1, and augmented their plasma membrane levels (Fig. 1). Hansen *et al*. also observed an increase in the amount of caveolin at the plasma membrane in syndapin2-siRNA-treated HeLa [17]. A possible explanation for the enhancement of EHD2/caveolin1 at the plasma membrane in syndapin2-depleted cells is that loss of caveolar pit formation by sydnapin2 deters internalization of EHD2/caveolin.

Interestingly, syndapin2 knockdown also enhanced the level of PIP2 at the plasma membrane (Fig. S1). Syndapin2 binds to synaptojanin [48], which converts PIP2 into PI4P. Furthermore, synaptojanin-catalyzed PIP2 hydrolysis occurs more efficiently on curved membranes generated by BAR domain-containing proteins, such as syndapin2 [49]. Thus, both the absence of curved caveolar membranes as well as diminished synaptojanin recruitment may increase PIP2 levels in syndapin2-depleted cells. Importantly, the finding that both PIP2 and EHD2 are increased at the plasma membrane of syndapin2-depleted cells fits with our model that PIP2 binding regulates EHD2 subcellular localization.

Using live cell imaging, Stoeber *et al*. found that caveolin1 moved along retracting actin filaments in cytochalasin D-treated cells. However, when EHD2 was depleted, caveolin1 no longer localized along actin filaments, suggesting that EHD2 provides a crucial link between caveolae and the actin cytoskeleton [19]. Our data suggest that it is through binding to PIP2 that EHD2 connects caveolae to actin microfilaments. This is likely given EHD2’s lipid-binding properties and lack of a known direct actin-binding domain. Additionally, EHD2-interacting proteins may contribute to the tethering of caveolae to actin. Indeed, we show that caveolin1 is dispersed across the plasma membrane in EHBP1-depleted cells (Fig. 1G), suggesting that EHBP1 may cooperate with EHD2 in linking caveolae to actin microfilaments.

**Figure 9:**
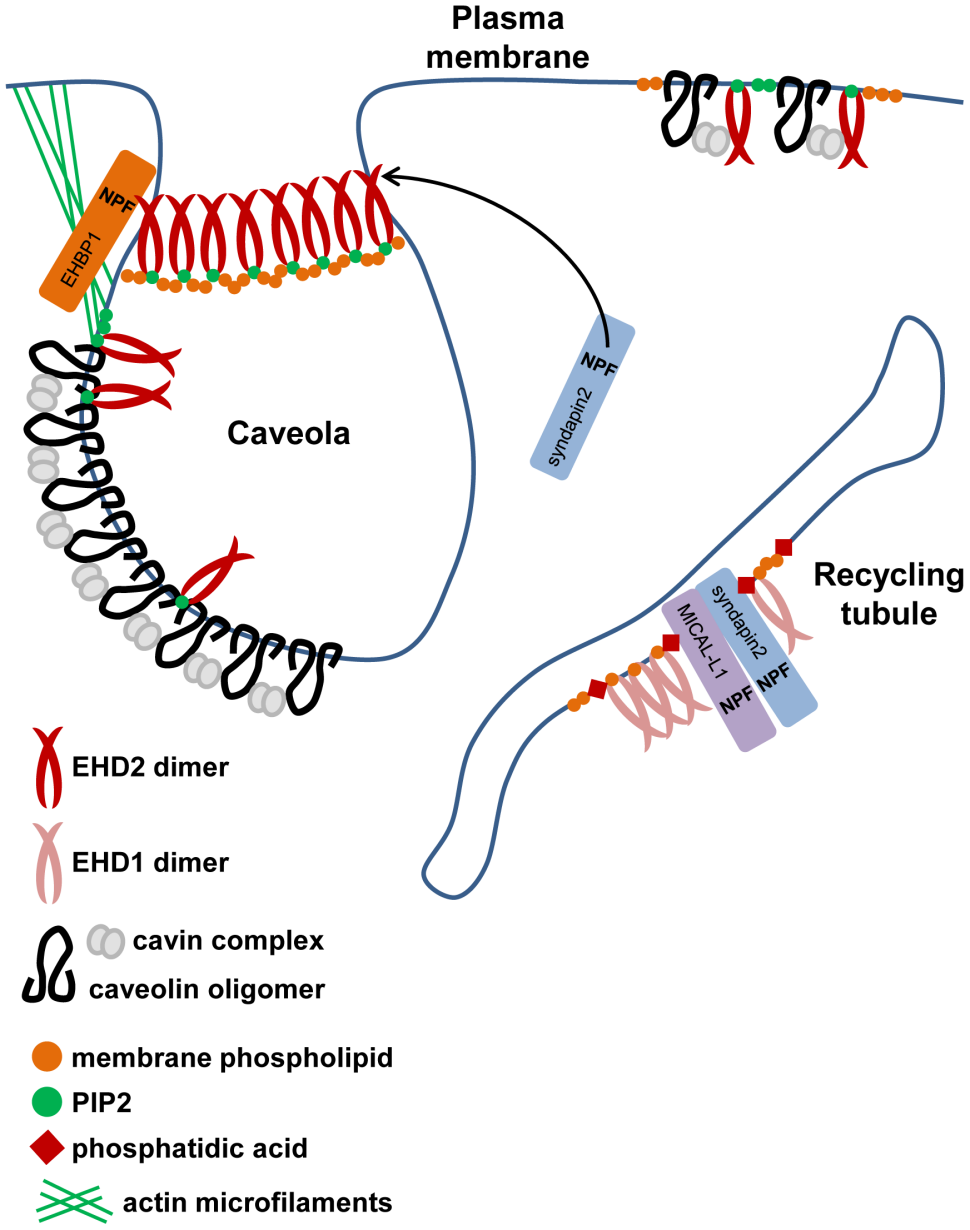
Model of EHD2 and EHD1 membrane localization. EHD2 localizes to the plasma membrane, and is predicted to form large oligomers at the neck of caveolae. PIP2, which is enriched in plasma membrane domains, including caveolae, has a crucial role in maintaining EHD2 at the cell surface. In the absence of caveolar invaginations, EHD2 maintains its plasma membrane association, likely through binding to PIP2 and, potentially, protein interactions mediated outside of the EH2 domain. EHD2 binds to the F-BAR domain-containing protein syndapin2, which facilitates membrane curvature, and also to EHBP1, which connects to the actin cytoskeleton. In contrast, localization of EHD1 to tubular recycling endosomes depends on binding of the EH1 domain to MICAL-L1 and sydnapin2, which require phosphatidic acid for their recruitment to the tubular recycling endosomes.

The mechanism by which EHD2 is recruited and maintained at the plasma membrane, and more specifically on caveolae, is likely multifaceted. Our studies indicate that binding of EHD2 to PIP2 contributes to EHD2’s plasma membrane localization and, potentially, to the tethering of caveolae to the actin cytoskeleton. In addition to lipid binding, protein interactions mediated through sites other than the EH domain may influence EHD2 localization. Potential candidates include caveolin and/or cavin. Indeed, recombinant EHD2 could pull down cavin1 from lysates [18]. Taken together, our results support a model in which EHD2 membrane localization is regulated by PIP2 rather than through canonical EHD protein interactions (Fig. 9). Thus PIP2 would be required for the localization of EHD2 to the plasma membrane and caveolae, where it eventually oligomerizes into large ring-like structures [18] and can interact with EHBP1 and syndapin2. In contrast, EHD1 subcellular distribution relies on the NPF motif-containing proteins MICAL-L1 and syndapin2, and their association with membrane phospholipids, such as PA [47] (Fig. 9). Thus, from these and previous studies, we can begin to build a model for the differential regulation of EHD subcellular localization and function.

## Materials and Methods

### Cells, Reagents and Antibodies

The HeLa cervical cancer cell line (ATCC-CCL2; Manassas, VA) was maintained in high glucose Dulbecco’s Modified Eagle’s Medium supplemented with 10% (v/v) fetal bovine serum (Sigma Aldrich), 200 U/ml penicillin, 200 µg/ml streptomycin, 2 mM L-glutamine, and 10 mM HEPES. All media reagents, except for the fetal bovine serum, were from Invitrogen. For siRNA-mediated gene knockdown, cells were transfected with 100 nM non-targeting control-, syndapin2- or EHBP1-siRNA (ON-TARGETplus SMARTpool siRNA from Dharmacon) using Oligofectamine (Invitrogen). Cells were analyzed 72 h after transfection with siRNA.

The phospholipase D inhibitors CAY10593 and CAY10594 were from Cayman Chemicals. Leupeptin and cytochalasin D were from Calbiochem. Lactacystin was from Andwin Scientific. Alexa-Fluor-488-conjugated human transferrin (transferrin-488) was from Molecular Probes.

The following primary antibodies were used in this study: affinity-purified rabbit polyclonal peptide antibody directed against the C-terminus of EHD2 (VERGPDEAMEDGEEGSDDEA) (AnaSpec), rabbit anti-syndapin2 (ProteinTech, #10518-2-ap, 1∶500 for immunoblot), rabbit anti-EHBP1 (Novus, #nbp1-93614, 1∶700 for immunoblot), rabbit anti-caveolin1 (Cell Signaling Technology, #3238, 1∶300 for immunofluorescence, 1∶1000 for immunoblot), mouse anti-pan actin (Novus, #nb600-535, 1∶2500 for immunoblot), mouse anti-cytochrome C (BD Pharmingen, #556433, 1∶400 for immunoblot), rabbit anti-GST (Millipore, #ab3282, 1∶200 for immunofluorescence), rabbit anti-Myc (Santa Cruz Biotechnology, #sc-789, 1∶200 for immunofluorescence), mouse anti-Myc (Invitrogen, #13-2500, 1∶200 for immunofluorescence), and mouse anti-HA.11 (Covance, #mms-101p, 1∶500 for immunofluorescence). Secondary antibodies used for immunoblotting were horseradish peroxidase (HRP)-conjugated goat anti-mouse IgG (H+L) (Jackson ImmunoResearch) and HRP-conjugated donkey anti-rabbit IgG (GE Healthcare Life Sciences). Secondary antibodies used for immunofluorescence were Alexa-Fluor-568-conjugated goat anti-rabbit IgG (H+L), Alexa-Fluor-568-conjugated goat anti-mouse IgG (H+L), and Alexa-Fluor-488-conjugated goat anti-rabbit IgG (H+L) (Molecular Probes).

### Recombinant DNA Constructs, Transfection and Protein Purification

Cloning of GFP-Myc-EHD1 was previously described [2]. Cloning of GFP-Myc-EHD2 and Myc-EHD2 was done similarly to the respective EHD1 constructs as described by Caplan *et al*. [2]. To generate GFP-EHD2-EH1, full-length EHD2 cDNA subcloned into the EGFP-C1 vector was truncated after the codon for amino acid 378, and cDNA encoding amino acids 379–534 of EHD1 was inserted. For GFP-EHD1-EH2, full length EHD1 in EGFP-C1 was truncated after the codon for amino acid 378, and cDNA encoding amino acids 379–543 of EHD2 was inserted. Myc-synaptojanin2 was a gift from Dr. M Symons (Feinstein Institute for Medical Research). Cloning of Myc-Vam6 was previously described [43]. HA-Arf6Q67L was kindly provided by Dr. J Donaldson (NHLBI, National Institutes of Health). HeLa cells were transfected using 6 µl X-tremeGENE 9 (Roche Applied Science) or 6 µl Lipofectamine 2000 (Invitrogen) and 2 µg DNA according to the manufacture’s protocol.

The cDNA encoding GST-PH-PLCδ1 in the pGEX-2T vector was generously provided by Drs. GR Hammond and T Balla (NICHD, National Institutes of Health). To purify GST-PH-PLCδ1, single colonies of BL21 cells (EMD Millipore) transformed with GST-PH-PLCδ1 cDNA were grown overnight at 37°C in 2X YT media (1.6% (w/v) tryptone, 1% (w/v) yeast extract, 0.5% NaCl, 50 µg/ml ampicillin). This overnight culture was used to inoculate 500 ml of 2X YT media containing 50 µg/ml ampicillin, and the bacteria were grown at 37°C with shaking until an optical density at 600 nm of 0.6–1.5 was reached. Protein expression was then induced with isopropyl β-D-thiogalactoside (1 mM final concentration) (Invitrogen). After shaking at 37°C for 3 h, the culture was centrifuged at 3000×g, and the pellet was washed twice with ice cold TBS. The cell pellet was resuspended in TBS containing 1 mM EDTA, 0.1% (v/v) 2-mercaptoethanol, and freshly added protease inhibitor cocktail (Roche Applied Science), and the cells were lysed using a French press. Following lysis, Triton X-100 was added to a final concentration of 1% (v/v), and the lysate was centrifuged at 18,000×g for 20 min to remove insoluble material. The cleared lysate was added to 2.5 ml of packed glutathione resin (GenScript) and incubated at 4°C overnight with rotation. The resin was washed twice with cold TBS, and the GST-tagged protein was eluted twice with 50 mM reduced glutathione in TBS (pH readjusted to 8.0). The eluates were pooled and dialyzed twice against buffer A (20 mM Pipes, pH 6.8, 137 mM NaCl, 2.7 mM KCl). The purified protein was quantified by the Pierce BCA Protein Assay (ThermoScientific) and by SDS-PAGE with Coomasie Brilliant Blue staining. Purified GST-PH-PLCδ1 was aliquoted and stored in 50% glycerol at −20°C.

### Immunofluorescence

Cells growing on coverslips were fixed with 4% (v/v) paraformaldehyde in PBS for 10 min, washed and incubated for 1 h at room temperature with primary antibody diluted in a staining solution of 0.2% (w/v) saponin and 0.5% (w/v) bovine serum albumin in PBS. After washing with PBS, the cells were incubated for 30 min at room temperature with fluorochrome-conjugated secondary antibody diluted in staining solution. To visualize filamentous actin, Alexa-Fluor-488-conjugated phalloidin (phalloidin-488) (Molecular Probes) was added at 1∶700 to the secondary antibody staining solution. Finally, the cells were washed and mounted onto slides with Fluoromount-G (SouthernBiotech). All images were acquired using a Zeiss LSM 5 Pascal confocal microscope (Carl Zeiss) by using a 63X, 1.4 numerical aperture objective with appropriate filters.

To visualize PIP2, cells were stained with purified GST-PH-PLCδ1 as described by Hammond *et al*. [37]. Cells growing on coverslips were fixed for 15 min at room temperature with 4% (v/v) paraformaldehyde and 0.2% (v/v) glutaraldehyde in PBS, and then washed 3X with 50 mM NH_4_Cl in PBS. After the washes, the cells were chilled by addition of ice cold buffer A and incubation at 4°C for 5 min. All subsequent steps were done at 4°C using pre-chilled buffers. Purified GST-PH-PLCδ1 was diluted to a final concentration of 100 nM in a solution of buffer A containing 5% (v/v) normal goat serum, 0.5% (w/v) saponin, and 50 mM NH_4_Cl. Cells were incubated with GST-PH-PLCδ1 for 45 min, and then washed twice with buffer A. Next, the cells were incubated for 1 h with rabbit anti-GST antibody diluted in a solution of buffer A containing 5% (v/v) normal goat serum and 0.1% (w/v) saponin. After two washes with buffer A, fluorochrome-conjugated secondary antibody and phalloidin-488 were applied in buffer A containing 5% (v/v) normal goat serum and 0.1% (w/v) saponin for 45 min. The cells were washed 4X with buffer A, and fixed with 2% (v/v) paraformaldehyde for 10 min at 4°C and an additional 5 min at room temperature. Finally, the cells were washed 3X with room temperature 50 mM NH_4_Cl in PBS, rinsed once with distilled water, and mounted onto slides with Fluoromount-G.

### Cell Lysis, Immunoblotting and Membrane Fractionation

To prepare lysates, cells were washed twice with PBS, harvested and resuspended in buffer containing 50 mM Tris, pH 7.4, 150 mM NaCl, 1% NP-40, 0.5% sodium deoxycholate, and freshly added protease inhibitor cocktail. The cell lysates were incubated on ice for 15 min and centrifuged to remove nuclear material. Protein level in the lysate supernatants was quantified using the Bio-Rad Protein Assay. Volumes of the lysate supernatants containing equal amounts of protein were mixed with 4X loading buffer containing 2-mercaptoethanol. The samples were heated at 100°C for 5 min, separated by SDS-PAGE, and transferred to nitrocellulose membranes (Bio-Rad). Membranes were blocked for 30 min at room temperature with 5% (w/v) dry milk in 0.3% (v/v) Tween-20 in PBS (PBST). After blocking, the membranes were incubated overnight at 4°C with primary antibody diluted in 5% dry milk in PBST. The next day, membranes were washed 3X with PBST, and incubated at room temperature for 45 min with HRP-conjugated secondary antibody diluted in PBST. Finally, the membranes were washed 3X with PBST, incubated with enhanced chemiluminescence substrate (ThermoScientific), and exposed to film (GeneMate).

Separation of membrane and cytosol fractionations by ultracentrifugation was performed as previously described [6]. Briefly, cells were homogenized on ice by 30 vertical strokes in a glass Dounce homogenizer in buffer containing 25 mM HEPES, 100 mM NaCl, 1 mM EDTA, pH 7.4 and freshly added protease inhibitor cocktail. The homogenized cells were centrifuged at 1000×g for 12 min to remove unbroken cells and nuclear material, and the supernatant was then ultracentrifuged at >100,000×g for 1 h. The supernatant (cytosolic fraction) was removed to a fresh tube, and the pellet (total membrane fraction) was dissolved in urea buffer (70 mM Tris-HCl, pH 6.8, 8 mM urea, 10 mM n-ethylmalemide, 10 mM iodoacetamide, 2.5% SDS and 0.1 mM DTT) by incubating at 37°C for 15 min. The entire membrane fraction and 7.5% of the cytosolic fraction were separated by SDS-PAGE and immunoblotted as described above.

## Supporting Information

Figure S1 **Syndapin2-siRNA increases PIP2 levels.** HeLa cells growing on coverslips were treated with non-targeting control- or syndapin2-siRNA for 72 h. The cells were fixed and stained with purified GST-PH-PLCδ1 to visualize PIP2 as described in the Materials and Methods. Cells were then analyzed by confocal microscopy. Images are z-series stacks. Bar, 10 µm. The confocal micrographs are representative of three independent experiments.
(TIF)

Figure S2 **Myc-synaptojanin2 expression dramatically decreases PIP2 levels.** HeLa cells transfected with Myc-synaptojanin2 were fixed and stained with anti-Myc antibody (A) and with purified GST-PH-PLCδ1 (to visualize PIP2) (B). The cells were subsequently stained with anti-GST antibody and fluorochrome-conjugated secondary antibodies, and assessed by confocal microscopy. The image showing PIP2 staining is a z-series stack. Bar, 10 µm. The confocal micrographs are representative of three independent experiments.
(TIF)
