# Supplementary figures and images for: Role of Phosphatidylinositol 4,5-Bisphosphate in Regulating EHD2 Plasma Membrane Localization
Source: PLoS One. 2013 Sep 10;8(9):e74519. doi: 10.1371/journal.pone.0074519 (PMC3769341; doi:10.1371/journal.pone.0074519)

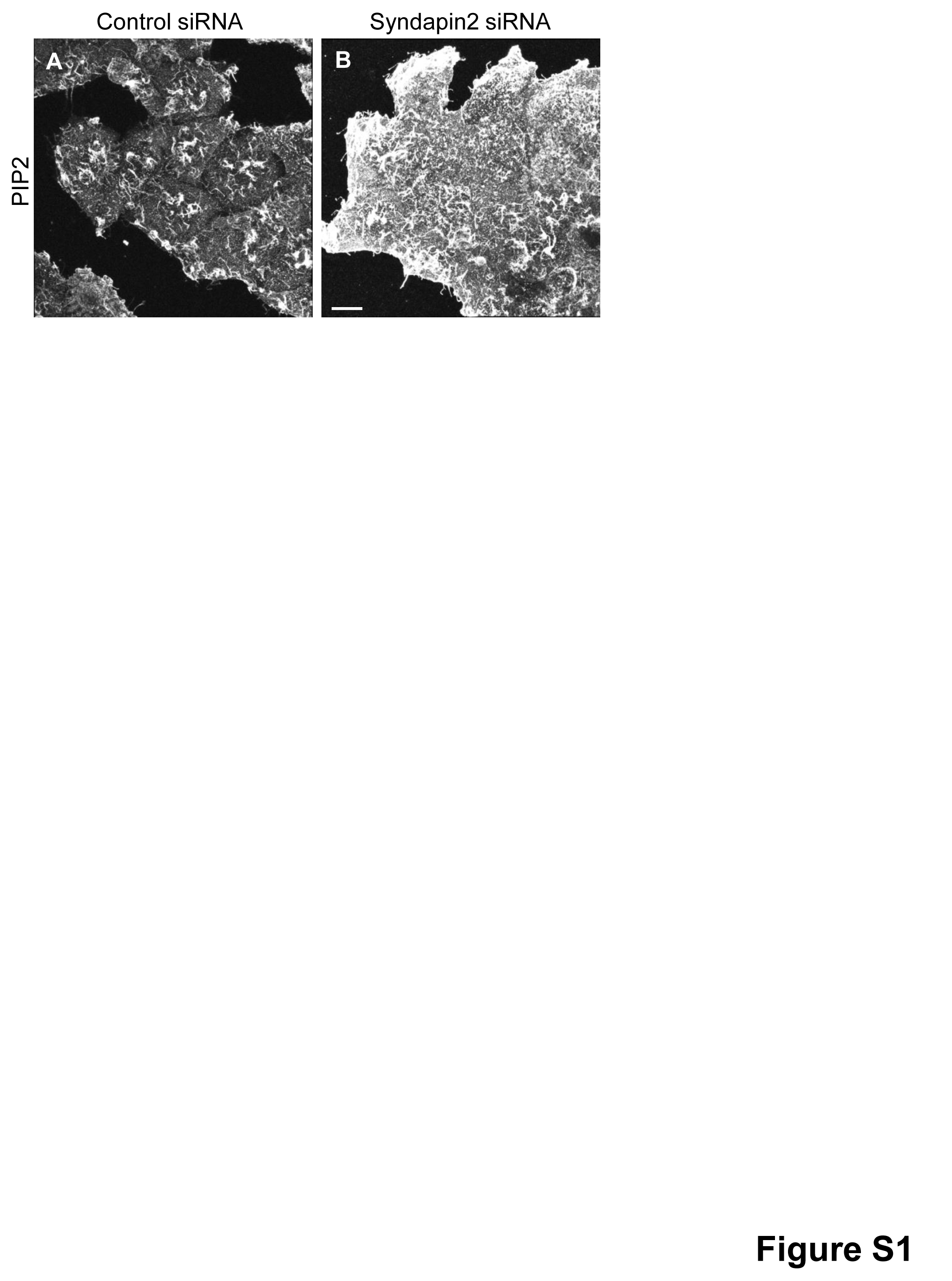

Supplement: Figure S1 — Syndapin2-siRNA increases PIP2 levels. HeLa cells growing on coverslips were treated with non-targeting control- or syndapin2-siRNA for 72 h. The cells were fixed and stained with purified GST-PH-PLCδ1 to visualize PIP2 as described in the Materials and Methods. Cells were then analyzed by confocal microscopy. Images are z-series stacks. Bar, 10 µm. The confocal micrographs are representative of three independent experiments. (TIF) [file pone.0074519.s001.tif]

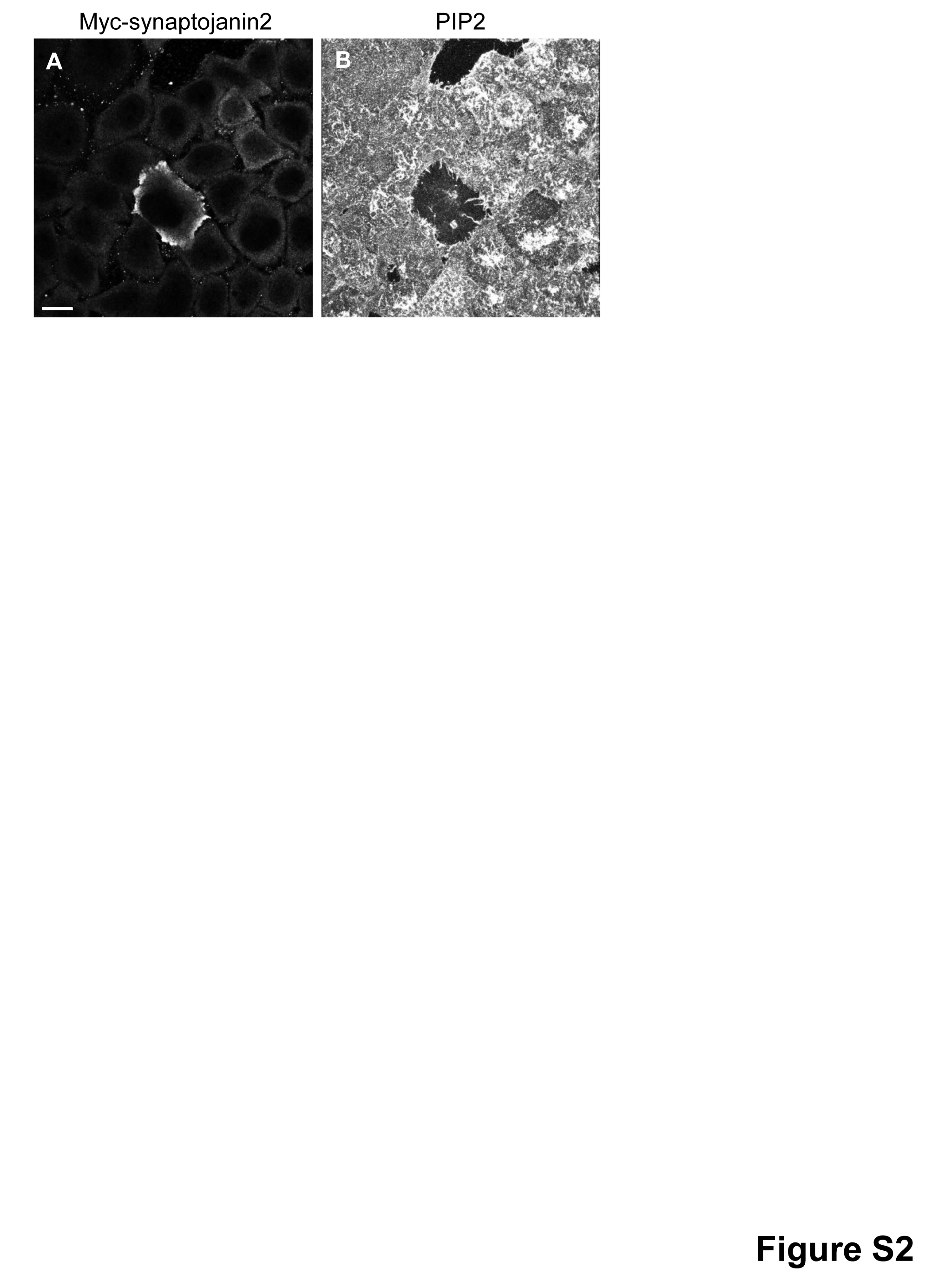

Supplement: Figure S2 — Myc-synaptojanin2 expression dramatically decreases PIP2 levels. HeLa cells transfected with Myc-synaptojanin2 were fixed and stained with anti-Myc antibody (A) and with purified GST-PH-PLCδ1 (to visualize PIP2) (B). The cells were subsequently stained with anti-GST antibody and fluorochrome-conjugated secondary antibodies, and assessed by confocal microscopy. The image showing PIP2 staining is a z-series stack. Bar, 10 µm. The confocal micrographs are representative of three independent experiments. (TIF) [file pone.0074519.s002.tif]
